# Beyond Antiretroviral Therapy: Molecular and Immunological Innovations in HIV Treatment

**DOI:** 10.3390/tropicalmed11050114

**Published:** 2026-04-26

**Authors:** Awadh Alanazi, Mohamed N. Ibrahim, Mohamed A. Elithy

**Affiliations:** 1 Department of Clinical Laboratory Sciences, College of Applied Medical Sciences, Jouf University, Sakaka 72388, Saudi Arabia; 2 Department of Clinical Laboratories Sciences, College of Applied Medical Sciences at Al Qurayyat, Jouf University, Al Qurayyat 77454, Saudi Arabia; mnabil@ju.edu.sa; 3 Department of Biochemistry, Ain Shams University, Cairo 11544, Egypt

**Keywords:** HIV-1, latency reversal, CCR5, broadly neutralizing antibodies, immune checkpoints

## Abstract

Despite prolonged viral inhibition with combination antiretroviral therapy (ART), HIV-1 survives as genetically intact, replication-capable proviruses within durable CD4+ T-cell fractions, involving central memory, transitional memory, and stem cell-like memory populations, as well as within tissue-resident compartments including lymphoid follicles and gut-associated lymphoid tissue. Reservoir stability is preserved via clonal growth of infected cells and epigenetic processes that impose proviral transcriptional silencing. As a result, current therapeutic approaches seek to either directly alter proviral survival or to improve immune-driven elimination of infected cells. At the molecular level, investigational strategies such as CRISPR–Cas9 and CRISPR–Cas12 gene-editing systems are intended to remove or induce inactivating mutations inside embedded proviral DNA, as well as alter host entrance co-receptors such as CCR5 to provide cellular resistance to infection. In addition, pharmacologic latency regulation is being studied via histone deacetylase inhibitors, protein kinase C agonists, and bromodomain inhibitors to reverse latency, along with Tat inhibitors and other transcriptional repressors aimed to persistently silence proviral expression. Moreover, immunological techniques aim to counteract inefficient endogenous antiviral defenses. Broadly neutralizing antibodies with tailored Fc-driven effector functions are under examination for both neutralization and antibody-dependent cellular cytotoxicity. Therapeutic vaccine approaches seek to elevate polyfunctional HIV-specific CD8+ T-cell responses, while adoptive cellular approaches, involving CAR-T cells aiming HIV envelope epitopes, remain in early clinical research. Immune checkpoint blockade is also being investigated to reverse T-cell depletion inside reservoir-rich tissues. Nevertheless, the key obstacles continue to be the diverse reservoir composition, restricted tissue penetration, viral escape, and safety limitations. The molecular and translational obstacles that characterize attempts toward an HIV cure must be addressed through ongoing multidisciplinary research.

## 1. Introduction

The development of combination antiretroviral medication in the middle of the 1990s significantly changed the course of HIV-1 infection from an inevitably fatal illness to a chronic condition that could be controlled [1,2]. A large proportion of patients adhering to modern ART regimens experience long-lasting virological suppression, which restores nearly normal life expectancy and stops further transmission. ART does not, however, entirely eliminate HIV-1; rather, it inhibits viral replication while preserving a reservoir of latently infected cells that continue to exist even after decades of intervention. Viral rebound happens almost universally quickly after therapy termination, usually in two to four weeks, requiring lifelong adherence to ART. This necessity enforces significant burdens such as medication toxicity, drug–drug interactions, expense, stigma, and the psychological effect of chronic illness management [3,4]. The existence of HIV-1 despite successful ART indicates the development of latent viral reservoirs during the initial phases of infection. These reservoirs are made up of long-lived cells that contain proviral DNA incorporated into the host genome, which are transcriptionally silent but capable of replication. The main cellular reservoir is made up of resting memory CD4+ T lymphocytes, which can survive for decades due to clonal expansion and homeostatic proliferation [5,6]. Furthermore, tissue-resident macrophages, microglia in the central nervous system, and other myeloid lineage cells lead to viral persistence in anatomically privileged regions with restricted ART entry and immune monitoring. The stability of the latently infected CD4+ T-cell reservoir indicates the extended nature of these cells and restricts the ability of antiretroviral treatment alone to eradicate HIV. As a result, efforts to either eradicate latent reservoirs (sterilizing cure) or permit long-term ART-free viral remission through immune-associated control (functional cure) have taken center stage in the pursuit of an HIV cure. In particular, a few cases of long-term HIV remission after allogeneic stem cell transplantation have provided clinical evidence that HIV can be cured. These individuals exhibit persistent antiretroviral therapy-free remission, undetectable plasma HIV RNA, and no replication-competent virus in blood or tissue reservoirs, indicating significant viral reservoir depletion, as presented in recent analyses [7]. Recent years have seen significant advancements in both molecular and immunological methods to address this challenge. However, the majority of currently explored cure-oriented strategies—such as immunological interventions like broadly neutralizing antibodies, latency-reversing techniques like “shock and kill,” and latency-promoting techniques like “block and lock”—primarily seek to alter the latent reservoir’s behavior or improve immune-mediated control. As a result, these tactics should be regarded as intermediate therapy approaches, since they may help reduce the reservoir or induce viral remission but are not yet able to consistently eradicate HIV on their own [8].

The past few years have seen a tremendous advancement in molecular techniques, mostly owing to advancements in nucleic acid-based technology and gene editing. Specifically, CRISPR-Cas systems have made it possible to target viral and host genomic regions precisely. Clinical observations in people with naturally occurring CCR5Δ32 mutations favor host-directed strategies, such as inactivation of the CCR5 co-receptor, which may be able to impart resistance to HIV entrance [9]. Parallel attempts concentrate on direct proviral attack, involving excision, fragmentation, or functional inactivity of integrated HIV DNA. The translational capacity of these methods has been reinforced by advancements in editing efficiency, guide RNA specificity, and delivery platforms; nonetheless, issues with viral genetic diversity and in vivo targeting are still being investigated [9,10].

Besides genome editing, the regulation of HIV transcriptional function has developed as a complementary molecular method. By inducing the regulated reactivation of silent proviruses, latency-reversing techniques aim to expose infected cells to viral cytopathic effects or immune clearance. These tactics employ drugs that affect chromatin shape, transcription factor recruitment, or intracellular signaling pathways driving HIV gene expression. By strengthening long-lasting epigenetic suppression of proviral transcription, latency-promoting or “block-and-lock” strategies, on the other hand, seek to lower the likelihood of spontaneous reactivation even in the absence of ART. Both paradigms are based on well-established molecular pathways and are still being improved through preclinical and early clinical testing [11,12].

In conjunction with molecular developments, immunological techniques have undergone tremendous refinement and currently play a major part in HIV cure research. Targeting conserved epitopes of the HIV-1 envelope glycoprotein, broadly neutralizing antibodies (bNAbs) have shown remarkable effectiveness and breadth against a variety of viral strains. In addition to direct neutralization, bNAbs activate Fc-mediated effector processes, such as phagocytosis and antibody-dependent cellular cytotoxicity, which may aid in the eradication of infected cells. Clinical research has demonstrated that bNAbs have dual antiviral and immunomodulatory capability by suppressing viremia, delaying viral rebound after ART discontinuation, and modulating host immunological responses [13,14]. But according to recent clinical research, viral comeback usually happens when antibody levels drop or resistant strains appear. As a result, while bNAbs can improve immune-mediated HIV infection management, they are more likely to support combined cure approaches than to achieve long-lasting viral eradication when used alone.

The goal of therapeutic vaccination approaches is to increase endogenous HIV-specific immunity, especially cytotoxic CD8+ T-cell responses that can identify and eradicate infected cells. Compared to previous therapeutic vaccine initiatives, improvements in antigen design, vector platforms, and immunization techniques have increased the strength, range, and longevity of vaccine-induced responses [15,16]. Simultaneously, adoptive cellular treatments have become a highly inventive extension of immunological intervention. The cytolytic ability and endurance of T cells are combined with precise antigen specificity in chimeric antigen receptor (CAR) T cells that have been modified with HIV-specific recognition domains produced from bNAbs [17,18]. Although clinical implementation is still in its early stages, preclinical research shows that these cells can identify HIV-infected sites, survive in vivo, and apply antiviral pressure.

Importantly, mounting data suggest that no single immunological or molecular treatment is likely to produce long-lasting ART-free remission on its own. Molecular strategies may change proviral transcriptional competency or decrease reservoir size, but they depend on efficient immune systems to eradicate any remaining infected cells. On the other hand, immunotherapies may be restricted by profoundly latent infection and require adequate antigen exposure. Therefore, the most likely route to a functional cure is increasingly thought to be a rational combination of tactics that combine immune-based treatments with molecular reservoir-targeting methods. Such methods may offer complementary advantages; molecular techniques may diminish or disrupt the viral reservoir, whilst immunological therapies may aid in the eradication of infected cells that become detectable. Nonetheless, there are still a number of biological obstacles to overcome, such as the uneven distribution of HIV reservoirs throughout tissues, inadequate latency reversal, and restricted immune effector function in persistent infection. This review summarizes current breakthroughs in molecular and immunological technologies in HIV treatment. We critically assess the mechanistic justification, recent preclinical and clinical data, and translational difficulties, with focus on how integrated treatment approaches may enable long-lasting viral control beyond traditional ART.

### Search Strategy and Selection Criteria

Systematic searches of PubMed, Web of Science, and Scopus were used to find pertinent material that looked at molecular and immunological approaches to HIV therapy and cure research. Combinations of “HIV reservoirs,” “latency reversal,” “CAR-T cells,” “CRISPR/Cas gene editing,” “broadly neutralizing antibodies,” and “immune checkpoint blockade” were among the search terms used. Peer-reviewed articles that were published between January 2010 and February 2026 were the main focus of the search. These studies included clinical, translational, and experimental research on molecular and immunological approaches that address HIV persistence. The exclusion criteria were non-English publications, records indexed in PubMed that were available only as conference abstracts without accessible full text, and articles unrelated to HIV treatment innovation.

## 2. HIV Biology and Therapeutic Barriers

### 2.1. Viral Reservoirs and Latency Mechanisms

The development of viral reservoirs and the maintenance of viral latency are the fundamental reasons why HIV persists despite long-term suppressive ART. Because of their long lifespan and capacity for homeostatic proliferation, resting memory CD4+ T cells are the most well-characterized and therapeutically significant reservoirs. These reservoirs are established very early during acute infection, frequently prior to the start of ART, and exhibit exceptionally slow decay kinetics under treatment. Although ART successfully inhibits active viral replication, it fails to address the integrated provirus in latently infected cells, allowing the reservoir to exist for decades [1]. The maintenance of reservoirs is controlled by complex molecular processes. Transcriptional silence results from the active recruitment of repressor complexes to the HIV LTR, such as Nurr1/NR4A2-mediated CoREST/HDAC/G9a/EZH2 complexes in microglia, and the restricted availability of important transcription factors (e.g., NF-κB, NFAT) in resting cells. Epigenetic repression through histone deacetylation, histone and DNA methylation, and incorporation into heterochromatin increases latency. Post-transcriptional barriers additionally limit viral RNA processing and protein generation. These interlocking systems work together to form a strong obstacle to HIV eradication [2].

### 2.2. Anatomical Reservoirs and Tissue Sanctuaries

HIV perseverance despite suppressive ART is additionally maintained by tissue sanctuaries and anatomical reservoirs that contain infected cells outside the peripheral blood compartment. The blood–brain barrier, which prevents many antiretroviral drugs and immune effector cells from penetrating the central nervous system (CNS), serves as a vital sanctuary. Microglia are the main cellular reservoir in the central nervous system (CNS), and HIV DNA has been found in brain tissue from people who are virally suppressed. Single-cell investigations have shown that both resident microglia and trafficking CD4+ T cells are infected CNS cells, and that both populations of clonally preserved T cells and microglia exhibit inflammatory transcriptional states. Crucially, regardless of systemic viral suppression, HIV transcription occurs in the brain, suggesting continuous low-level viral activity in this compartment [3]. The majority of the body’s HIV reservoir is found in the gut-associated lymphoid tissue (GALT), which is the biggest lymphoid organ system. During an acute infection, the GALT is specifically targeted, resulting in a significant reduction in CD4+ T lymphocytes in the intestinal mucosa. The gut is still a location of ongoing viral multiplication, immune activation, and reservoir maintenance even after ART is started. A great deal of HIV infection is shown by activated CD4+ T cells, especially CCR5+ and tissue-resident memory T cells, which are abundant in GALT. HIV infection and latency in CD4+ T cells, especially in CCR6+ subsets, can be increased by intestinal endothelial cells. The specific immunological milieu of the gut, characterized by persistent inflammation and microbial translocation, might lead to reservoir survival through homeostatic proliferation and immune activation [4]. The vaginal tract, bone marrow, and lymph nodes are other anatomical reservoirs. Lymph nodes comprise follicular helper T cells (TFH) within B-cell follicles, a region with restricted cytotoxic T lymphocyte (CTL) accessibility, enabling infected TFH cells to survive despite immune surveillance. Long-lived haematopoietic stem and progenitor cells found in the bone marrow may extend the life of the reserve. These various structural reservoirs require treatment approaches that can penetrate sanctuary sites and achieve wide tissue dissemination.

### 2.3. Immune Evasion Strategies

Antiretroviral therapy effectively suppresses plasma viremia, but HIV persists because the host immune system cannot eradicate all infected cells. Both innate and adaptive immunity are permanently compromised by HIV infection, and these deficiencies persist even with extended antiretroviral therapy. These flaws enable latently infected cells to endure and support lasting viral reservoirs [1]. One significant mechanism is the malfunctioning of HIV-specific CD8+ cytotoxic T cells (CTLs). T cell fatigue is a condition brought on by prolonged exposure to HIV antigens. Reduced production of cytokines, including interferon-γ and tumor necrosis factor-α, as well as decreased cytolytic activity, are characteristics of exhausted CTLs. The persistent expression of inhibitory receptors, such as cytotoxic T lymphocyte-associated antigen 4 (CTLA-4) and programmed cell death protein 1 (PD-1), is linked to this fatigue. These findings show that, even during ART, CTLs are less able to identify and destroy HIV-infected cells [5]. Another well-known method of viral escape is genetic diversity. HIV often acquires mutations in CTL-recognized epitopes. Despite the existence of immune responses directed against earlier viral sequences, these alterations lessen identification by particular CTLs, allowing infected cells to endure. HIV infection impairs the activity of natural killer (NK) cells, which also aid in immunological regulation. Modifications in activating and inhibitory receptor expression limit their potential to recognize and kill sick cells. Research indicates that these functional deficits may endure after ART, which would restrict the removal of remaining infected cells [6]. Lastly, prolonged viral persistence is indirectly influenced by ongoing inflammation and immunological activation. ART-treated patients have higher levels of inflammatory cytokines and more indicators of immunological activation. By fostering an environment that promotes cell survival, persistent inflammation may help maintain latent reservoirs and impede efficient immune surveillance [7].

**Figure 1:**
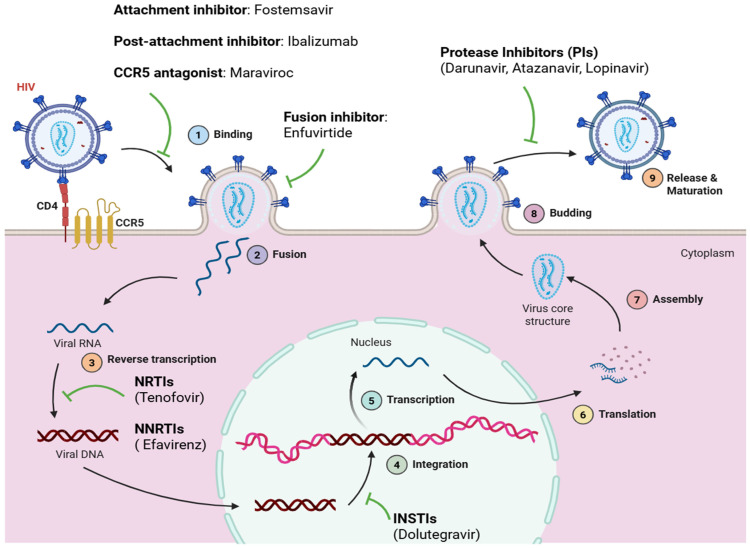
Approved antiretroviral drugs mapped across the HIV life cycle. Created with BioRender.com.

Even though HIV creates long-lived reservoirs that escape immune elimination, active viral replication remains dependent on a set of defined molecular steps that are efficiently attacked by current antiretroviral therapy ART. As shown in Figure 1, approved medicines disrupt the viral life cycle at several stages, such as entry, reverse transcription, integration, and maturation. Entry and fusion inhibitors halt infection of new cells, reverse transcriptase inhibitors inhibit the creation of viral DNA, integrase inhibitors stop genomic integration, and protease inhibitors interrupt the formation of mature infectious virions. Despite this multifaceted suppression of active replication, these treatments do not eradicate latent proviral DNA, enabling persistent reservoirs to appear as a significant obstacle to cure.

## 3. Molecular Innovations

### 3.1. Host-Directed Gene Editing Strategies

By altering host cells to prevent infection or interfering with host elements necessary for viral replication, host-directed gene editing techniques seek to achieve long-lasting HIV control. Targeting the CCR5 co-receptor is the strategy with the greatest level of validation. CCR5-tropic (R5) HIV-1 strains, which are more common during early infection and transmission, require CCR5 for entry. Strong genetic data for CCR5 as a therapeutic target is provided by the naturally occurring CCR5-Δ32 deletion, which provides resistance to HIV infection in homozygous people and delays illness progression in heterozygotes [8]. Numerous gene-editing technologies, such as CRISPR-Cas systems, zinc finger nucleases (ZFNs), and transcription activator-like effector nucleases (TALENs), have been created to mimic this protective behavior. Clinical testing for ZFNs in HIV-positive people was the first approach achieved. Ex vivo CCR5-disrupted autologous CD4+ T cells showed modest rises in CD4+ T cell numbers and acceptable safety in phase I trials. In analytical therapy interruption investigations, prolonged viral rebound was noticed in a portion of patients, especially those with heterozygous CCR5-Δ32 backgrounds. This suggests that while CCR5-edited cells can aid in viral suppression, they are ineffective as an independent treatment strategy [19].

Because of its effectiveness, programmability, and comparatively simple design, CRISPR-Cas9 has become the most popular gene-editing technology [9]. Preclinical research has shown effective CCR5 disruption in haematopoietic stem cells and CD4 T cells, providing resistance to HIV infection in humanized mouse models and in vitro. Under viral pressure, edited cells show a selective survival advantage, indicating the possibility of long-term survival. The HIV-1 counterpart of the EBT-101 initiative, which uses CRISPR-Cas9 delivered via adeno-associated virus (AAV) to target conserved HIV areas, has advanced to phase I clinical examination after demonstrating safety and wide biodistribution in nonhuman primates. Nevertheless, viral return upon treatment stoppage shows limits of CCR5 editing alone, involving survival of pre-existing infected cells and development of CXCR4-tropic variants [20].

**Figure 2:**
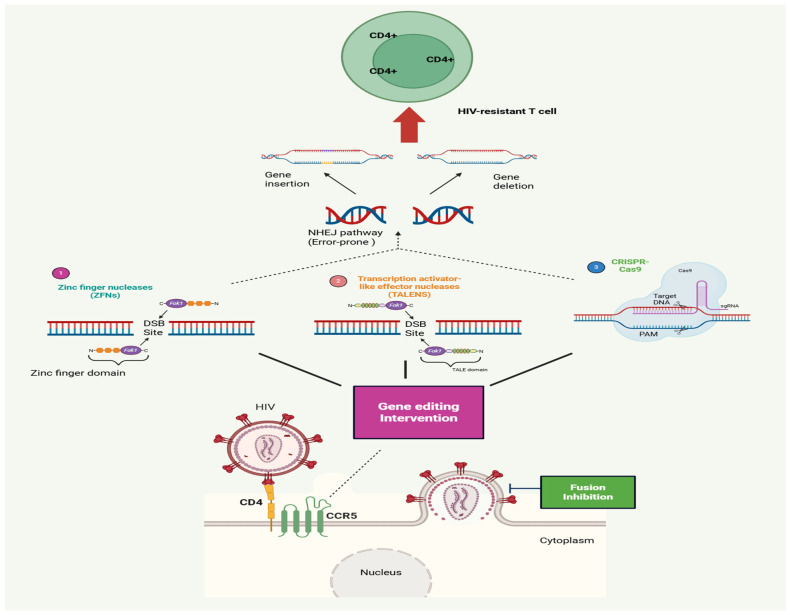
Gene-editing approaches for HIV therapeutic intervention. Created with BioRender.com.

Other host dependency factors have been investigated in addition to CCR5. CXCR4, the alternate co-receptor employed by X4-tropic strains, is a viable target but creates security issues due to its crucial involvement in haematopoiesis. The chromatin-binding protein LEDGF/p75, which binds HIV integrase to transcriptionally active genomic areas, has been the subject of other tactics [12,13,14,15,16,17,18,19,20,21]. It has been suggested that altering this interaction could reroute viral integration toward transcriptionally silent sites, promoting long-lasting proviral silence as opposed to eradication. These methods are still in the early phases of research despite encouraging preclinical results. (Figure 2).

### 3.2. Direct Targeting of Integrated Provirus

One method of eliminating HIV reservoirs that is mechanistically straightforward is direct targeting of integrated proviral DNA. It is possible to develop gene-editing tools like CRISPR-Cas9 and TALENs to identify conserved areas of the HIV genome, causing double-strand breaks that are fixed by error-prone non-homologous end joining. This approach introduces insertions or deletions that alter viral genes, while dual targeting of the long terminal repeats (LTRs) around the provirus can eliminate the whole integrated viral genome. Proof-of-concept investigations have shown effective proviral removal and inactivation in cell culture systems, primary CD4+ T cells, and animal models. CRISPR-mediated breakage of HIV-1 provirus stops viral reactivation in latently infected cells and decreases viral rebound in humanized mouse models [11,12,13,14,15,16,17,18,19,20,21,22]. Combination approaches focusing on both viral sequences and CCR5 have shown increased effectiveness compared with single-target strategies. Particularly, CRISPR-based proviral excision in conjunction with long-acting slow-effective release antiretroviral treatment led to the eradication of detectable HIV infection in a fraction of humanized mice, highlighting the significance of preventing fresh rounds of infection during genome editing [23,24]. The most cutting-edge translational endeavor in direct proviral targeting is the EBT-101 clinical study. Preclinical research with SIV-infected macaques showed significant decreases in intact proviral DNA and widespread tissue biodistribution, including the central nervous system and lymphoid organs [25]. Phase I clinical data in ART-suppressed patients demonstrated safety and tolerability, but inadequate reservoir eradication was indicated by viral resurgence after therapy interruption [26].

### 3.3. RNA-Based Molecular Therapies

RNA-based molecular treatments provide complementary methods for preventing HIV replication and regulating viral persistence by focusing on viral or host RNA rather than causing permanent genetic changes. These strategies involve RNA interference, antisense and splice-switching oligonucleotides, RNA-directed epigenetic silencing, and messenger RNA-based tools. Their reversibility and lack of genetic incorporation differentiate them from DNA-editing approaches and contribute to their advantageous safety profiles. The most thoroughly studied RNA-based strategy in HIV research is RNA interference (RNAi), which uses short hairpin RNAs (shRNAs) and small interfering RNAs (siRNAs). RNAi uses endogenous cellular pathways to orchestrate the translational suppression or sequence-specific destruction of HIV RNA transcripts. SiRNAs that target conserved areas of the HIV genome, such as tat, rev, gag, and pol, have been shown in several in vitro and ex vivo experiments to greatly reduce viral replication in infected CD4+ T cells and macrophages [27]. Furthermore, target cells’ vulnerability to HIV infection has decreased due to RNAi-mediated suppression of host dependence factors such as CCR5 and CXCR4. RNAi-based therapeutics offer a prospective adjunct or alternative to cART by allowing sequence-specific suppression of viral and host elements critical for HIV-1 replication, possibly addressing drug resistance and viral diversity. Preclinical and early clinical research validate their safety and durability, although effective delivery methods and preventing mutational escape continue to be major obstacles for clinical application [28].

ASOs, or antisense oligonucleotides, are an additional RNA-based treatment approach. Short synthetic nucleic acids known as ASOs attach complementary RNA sequences, causing RNase H-induced breakdown, translational repression, or RNA processing alteration. ASOs targeting HIV transcripts, such as regulatory areas such as the trans-activation response (TAR) element, have exhibited antiviral effectiveness in preclinical tests by suppressing Tat-mediated transcriptional activation. Phosphorothioate backbones, 2′-O-methyl substitutions, and locked nucleic acid alterations are examples of chemical modifications that have enhanced ASO stability, cellular absorption, and resistance to nuclease destruction [29].

A specific subclass of ASOs called splice-switching oligonucleotides is made to obstruct HIV RNA splicing. To produce several viral proteins from a single main transcript, HIV uses a sophisticated alternative splicing procedure, which must be precisely regulated for productive infection. By targeting particular splice donor or acceptor sites, splice-switching oligonucleotides prevent the synthesis of vital viral mRNA isoforms and hinder the creation of viral proteins. Preclinical research has shown that structural and regulatory protein expression can be decreased by modifying HIV RNA splicing. However, clinical testing of splice-switching approaches has been restricted by difficulties in attaining enough intracellular concentrations within reservoir cells, as well as by viral sequence diversity that may restrict sustained efficiency [30].

RNA-directed epigenetic silencing offers an extra method for prolonged viral control by imposing transcriptional inhibition of the integrated provirus. The siPromA system uses small RNAs aiming NF-κB binding sites within the HIV-1 promoter, causing transcriptional gene silencing via recruitment of repressive chromatin modifiers and chromatin remodeling. This method encourages a highly suppressed transcriptional state rather than eliminating the provirus [31]. In humanized mouse models, siPromA treatment led to lower HIV RNA expression, defense against infection, and delayed viral resurgence following treatment discontinuation. These results lend credence to the possibility of using RNA-directed epigenetic silencing as a component of a “block-and-lock” strategy that aims for long-term suppression as opposed to reservoir removal [32].

Messenger RNA-driven therapy delivered via lipid nanoparticle technologies has also been investigated for HIV applications, involving transient expression of widely neutralizing antibodies and vaccine immunogens. RNA-based treatments offer benefits like reversibility, lack of genomic modification, and the capacity to target viral and host transcripts that can be hard to modulate via traditional pharmacological techniques, despite ongoing delivery, stability, and specificity issues [33] (Table 1).

### 3.4. Pharmacological Manipulation

Pharmacological control of HIV latency continues to be an important focus of cure-centered research, as the survival of transcriptionally silent but replication-competent proviruses in durable cellular reservoirs represents the primary barrier to viral elimination under suppressive ART. Two essentially distinct pharmacological approaches have appeared to address this problem: “shock and kill,” which intends to cause viral gene expression in latently infected cells followed by their eradication, and “block and lock,” which aims to impose long-lasting transcriptional silencing of the provirus and thereby attain prolonged viral control without reservoir clearance. In order to trigger HIV transcription in resting CD4 T cells and other reservoir populations, the shock-and-kill method uses latency-reversing agents (LRAs) [42]. Once viral proteins are produced, infected cells are likely to become sensitive to immune-induced death or viral cytopathic effects, while ART inhibits de novo infection. Numerous types of LRAs with different mechanisms have been assessed. Histone deacetylase inhibitors (HDACis), involving vorinostat, panobinostat, and romidepsin, work by reversing repressive chromatin changes at the HIV long terminal repeat (LTR), thereby increasing access of transcriptional machinery. Clinical research has repeatedly demonstrated rises in cell-associated HIV RNA after HDACi administration, validating effective latency reversal in vivo. However, these elevations have not been accompanied by long-lasting decreases in reservoir size or slowed viral rebound during analytical treatment interruption, underscoring a crucial gap between transcriptional activation and reservoir eradication [43]. This disparity suggests that while HDAC inhibitors can trigger HIV transcription, reactivation by itself does not guarantee effective removal of infected cells in vivo.

Another family of LRA is represented by protein kinase C (PKC) agonists, which activate NF-κB and associated signaling pathways to enhance HIV transcription. Strong latency reversal is demonstrated by substances like bryostatin-1 and ingenol derivatives in both in vitro and ex vivo models utilizing cells from ART patients. However, systemic immune activation, dose-limiting toxicities, and no discernible effect on reservoir size have limited clinical application. These results imply that strong transcriptional activation may be counteracted by immunological activation and toxicity, which limit the therapeutic window of these drugs. By disrupting the connections between BET proteins and acetylated histones, bromodomain and extraterminal domain (BET) inhibitors modify HIV transcription by changing transcriptional control at the LTR. By triggering pro-inflammatory cytokine signaling and activating innate immunity pathways, toll-like receptor (TLR) agonists, especially those that target TLR7, indirectly reverse latency. They also have the potential to improve antiviral immune responses [42].

Despite thorough clinical testing, LRAs have revealed significant flaws in the shock-and-kill strategy. Only a small percentage of latently infected cells react to any given drug, resulting in incomplete and uneven latency reversal with existing therapies. Furthermore, after reactivation, viral protein expression levels are frequently too low to initiate successful immune recognition. The functional reduction in HIV-specific CD8+ T cells linked to chronic HIV infection further restricts the removal of reactivated cells. As a result, rather than being eradicated, many cells that experience temporary reactivation may revert to latency. Collectively, these drawbacks show that effective latency reversal and mechanisms that improve immune-mediated removal of infected cells are necessary for the shock-and-kill technique to be applied successfully. Although clinical results have been modest thus far, these findings have highlighted the need to increase the “kill” component via combination techniques incorporating therapeutic vaccines, broadly neutralizing antibodies, or immune checkpoint inhibitors. Consequently, shock-and-kill tactics should not be seen as a stand-alone strategy that can achieve a full HIV cure, but rather as a way to disturb and possibly lower the latent reservoir.

The block-and-lock approach, on the other hand, attempts to stabilize rather than reverse HIV latency in order to achieve a functional cure. Even in the absence of ART, this strategy aims to transform the latent reservoir into a profoundly quiet state that is impervious to pharmacological reactivation or physiological stimulation. Block-and-lock therapies employ latency-promoting agents (LPAs) that maintain transcriptional inhibition via epigenetic mechanisms or by attacking viral regulatory proteins essential for effective gene expression [44]. Although sustaining deep latency may help maintain long-term viral control, this approach does not eradicate infected cells; therefore, it may lead to functional remission rather than total viral eradication. As a result, block-and-lock strategies are still under investigation and have mainly shown proof-of-concept in preclinical models, suggesting that more validation is necessary before they can be regarded as a viable pathway to long-term ART-free remission.

Didehydro-cortistatin A (dCA), a well-known example, suppresses the HIV Tat protein, which is a major factor in the transcriptional elongation of the virus. dCA promotes the formation of a deep latent state and reduces HIV transcription by blocking Tat–TAR interactions. Preclinical research in humanized mouse models and cellular systems has demonstrated that dCA can confer resistance to repeated LRA reactivation and postpone or prevent viral rebound following ART cessation. According to these results, Tat inhibition may cause proviral silence that is more persistent than that caused by traditional transcriptional inhibitors [45].

Curaxins, which affect chromatin architecture and encourage transcriptional suppression of the HIV LTR, and repurposed drugs like levosimendan, which have shown latency-promoting effects in animal models, are other LPAs that are being studied [46]. Furthermore, under some cellular contexts and dosage conditions, some BET inhibitors may have latency-stabilizing impacts; however, their dual ability to either promote or hinder HIV transcription calls for cautious consideration [47].

### 3.5. Novel Antiviral Targets Beyond Classical ART

Novel antiviral targets that may aid in treatment approaches have surfaced in addition to the conventional reverse transcriptase, protease, and integrase inhibitors. Targeting the HIV-1 capsid protein (CA), which has several crucial functions in the viral replication cycle, capsid inhibitors are a particularly promising class of drugs. Lenacapavir, the first-in-class capsid inhibitor authorized for use in clinical trials, adheres to the CA hexamer interface, affecting capsid assembly, stability, nuclear import, and uncoating. Because of its extended half-life, six-monthly subcutaneous administration is possible, providing the possibility of long-acting ART regimens to enhance adherence [48].

Capsid inhibitors may aid in curative strategies through different routes. They can stop the formation of new infections in cells with low-level viral replication by blocking nuclear import of the pre-integration complex. Targeting cell-to-cell transmission, which can happen even with antiretroviral therapy, may be made possible by their activity against mature capsid structures. Lenacapavir’s long-acting pharmacokinetics provide persistent viral suppression during reservoir-targeting therapy, making it appealing for use in conjunction with other curative approaches [49].

Another new class of drugs is maturation inhibitors, which work by stopping the Gag polyprotein from being cleaved during the last stage of viral assembly. Naturally existing mutations in the Gag cleavage site that impart resistance restricted the antiviral effectiveness of bevirimat, the first maturation inhibitor. Enhanced resistance profiles for second-generation maturation inhibitors are being developed. By stopping the generation of infectious virions from reactivated reservoir cells, these medicines may aid in cure methods and might lower the risk of viral rebound during latency reversal therapies [50].

Additional ways to stop the transmission of the virus include attachment and entry inhibitors that target the HIV envelope glycoprotein or host receptors. Multidrug-resistant viruses are susceptible to the action of fostemsavir, an attachment inhibitor that latches to gp120 and stops CD4 interaction [51]. Without reducing CD4+ T cells, the monoclonal antibody ibalizumab, which targets CD4, prevents viral entry [52]. By stopping the propagation of reactivated reservoir cells or the reinfection of altered cells, these medicines may be especially useful in combination treatment approaches.

## 4. Immunological Innovations Targeting HIV Control

### 4.1. Broadly Neutralizing Antibodies (bNAbs)

One of the most promising immunological developments for HIV treatment and cure-directed approaches outside of traditional ART is broadly neutralizing antibodies (bNAbs). A small percentage of HIV-positive people who experience exceptionally strong and widespread humoral immune responses following protracted infection are found to have these antibodies. About 10–30% of HIV-positive people naturally produce bNAbs, which usually requires years of constant viral exposure [53]. bNAbs neutralize genetically different HIV-1 strains by attacking conserved epitopes on the viral envelope glycoprotein (Env), which are important for viral entry and consequently subject to stringent functional restrictions.

On the basis of epitope specificity, numerous major classes of bNAbs have been identified through comprehensive antibody isolation and characterization studies. These involve antibodies concentrating on the CD4 binding site (VRC01, 3BNC117, N6), the V1/V2 apex region (PG9, PGT145, CAP256-VRC26), the V3 glycan supersite (PGT121, 10-1074), the gp120–gp41 interface (35O22, 8ANC195), and the membrane-proximal external region (MPER) of gp41 (10E8, LN01). Second-generation bNAbs with much increased potency and breadth, able to neutralize more than 90% of circulating HIV-1 strains at nanomolar or sub-nanomolar amounts, are the result of iterative advancements in antibody research, structural biology, and engineering [13].

Preclinical research in non-human primates and humanized mice models has shown that bNAbs can inhibit viral replication, postpone viral rebound after stopping antiretroviral therapy, and occasionally result in long-term virological control [53]. These investigations further demonstrated that bNAbs use Fc-dependent immune effector activities to mediate antiviral action beyond neutralization of free virions. In particular, bNAbs facilitate complement-dependent cytotoxicity (CDC), antibody-dependent cellular cytotoxicity (ADCC), and antibody-dependent cellular phagocytosis (ADCP), allowing the immune system to eradicate HIV-positive cells that display Env on the cell surface.

Quantitative proof-of-concept for the therapeutic use of bNAbs has been obtained through clinical trials. In viremic subjects not on ART, bNAb monotherapy reduced plasma viral load by 0.8 to 2.5 log_10_ copies/mL over the course of about 28 days, with both degree and duration of containment t dependent on baseline viral susceptibility to the administered antibody [14]. According to another study, bNAb 3BNC117 infusions during ATI considerably postponed viral resurgence in comparison to historical controls (median ~6.7 weeks following two infusions and up to ~9.9 weeks post four infusions). A number of individuals continued to be suppressed until their antibody levels decreased [54]. In a phase 1b trial, the bNAbs 3BNC117 and 10-1074 were administered in combination to individuals with viremic HIV-1 and were well tolerated. In subjects whose virus was susceptible to both antibodies, viral load decreased by about 2 log_10_ copies/mL and stayed suppressed for up to three months following infusion. Crucially, dual resistance did not develop, indicating that combined bNAb therapy may prevent viral escape [55]. Using the bNAb 3BNC117 at ART initiation accelerated viral load drop and decreased infected CD4+ T cells in comparison to ART alone, with larger effects in those whose virus was antibody-sensitive. HIV-specific CD8+ T-cell responses were also enhanced. These patients had a higher chance of maintaining ART-free viral control throughout therapy discontinuation [56].

**Figure 3:**
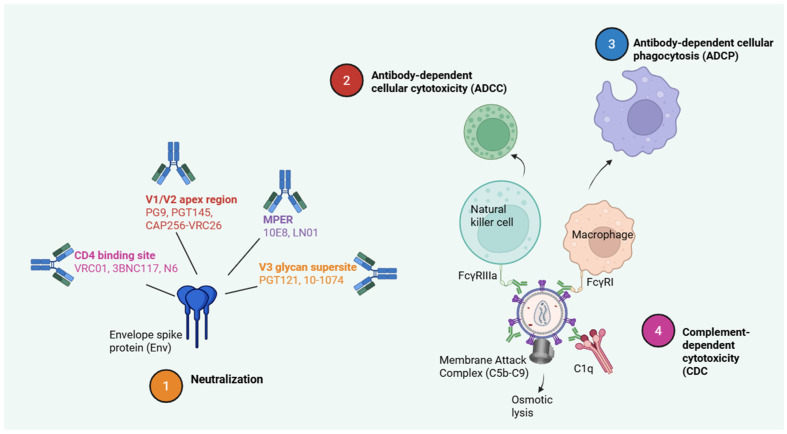
Mechanisms and roles of broadly neutralizing antibodies (bNAbs) targeting the HIV-1 envelope. Created with BioRender.com.

Because of their comparatively long serum half-lives (usually two to four weeks), bNAbs can be administered intravenously or, seldomly, subcutaneously. Fc engineering has additionally prolonged antibody persistence and boosted effector function. However, viral resistance can develop, especially during monotherapy, and pre-existing resistance warrants the adoption of antibody combinations with complementary breadth or viral susceptibility screening [15]. Despite obstacles relating to cost, delivery, and resistance, bNAbs remain a major immunological pillar in treatments aimed at sustainable HIV control and functional cure. (Figure 3).

### 4.2. Therapeutic HIV Vaccines

In order to achieve long-lasting immune-mediated viral control or help reduce the latent reservoir in the absence of ongoing ART, therapeutic HIV vaccines are intended to boost or reroute virus-specific immune responses in people with established infection. Unlike preventive vaccinations, therapeutic vaccination has to deal with long-lived viral reservoirs, T-cell depletion, and chronic immunological activation, all of which significantly reduce immune efficiency. The molecular basis for therapeutic immunization is derived from studies in elite controllers, who spontaneously keep plasma HIV RNA levels below detection without ART. These people typically show intact CD4+ T-cell assistance in addition to strong, polyfunctional HIV-specific CD8+ T-cell responses that target conserved viral epitopes. However, the majority of HIV-positive individuals have diminished cytotoxic capacity, growing immunological dysfunction, and viral escape mutations that impair antiviral immunity. The goal of therapeutic vaccinations is to either reorient immune responses toward conserved viral genome areas that are less susceptible to mutation or to restore these compromised immunological capabilities [16].

Numerous vaccination platforms have been assessed. Although HIV antigen-encoding DNA vaccines are scalable and safe, they usually cause very mild immune reactions in people. Adenovirus, vesicular stomatitis virus, modified vaccinia Ankara (MVA), and cytomegalovirus (CMV) vectors are examples of viral vector vaccines that typically elicit greater cellular immunity, especially when utilized in heterologous prime-boost regimens. Dendritic cell vaccines load autologous dendritic cells with HIV antigens ex vivo to improve antigen presentation and T-cell priming, whereas peptide-based vaccinations offer specific HIV epitopes and may attack conserved portions of the virus.

In several investigations, the Vacc-4x peptide vaccine, which is made up of HIV Gag p24 peptides, elicited T-cell responses unique to HIV. In a phase 1B/2A trial, therapeutic immunization with Vacc-4x and the latency-reversing drug romidepsin resulted in a ~38% decline in the replication-competent reservoir (IUPM) and a substantial decrease in total HIV-1 DNA in evaluable patients receiving suppressive ART. The majority of the side effects were modest, and the regimen was typically safe and well-tolerated. The limited reservoir reduction, however, suggests that more modification is required for a significant therapeutic impact [57]. In cynomolgus macaques, MHC-E-restricted CD8+ T-cell responses were generated by species-matched CMV vectors encoding SIV antigens. Following a challenge, about half of the vaccinated animals were able to control SIV, and protection was connected to an immunological signature linked to IL-15. The findings support CMV-based HIV vaccination methods by demonstrating that host-matched CMV vectors are necessary for this unusual protective response [58].

### 4.3. Cellular Immunotherapies

Cellular immunotherapies based on chimeric antigen receptors (CARs) are being investigated as immunological strategies to improve HIV infection management by rerouting cytotoxic immune cells onto HIV-infected targets. These strategies are based on well-established principles of adoptive cell treatment and are aimed at supplementing antiretroviral treatment by focusing on infected cells that display viral antigens. CAR-T cells are autologous T cells that have been genetically transformed to express an artificial receptor made up of intracellular signaling modules, a transmembrane region, and an extracellular antigen-recognition domain. In HIV cases, the recognition domain is usually derived from the CD4 molecule or broadly neutralizing antibodies (bNAbs) targeting HIV envelope glycoproteins. This design allows CAR-T cells to recognize infected cells expressing gp120 or gp41, overcoming viral immune escape methods such as Nef-mediated HLA class I downregulation. First-generation CARs with just the CD3ζ signaling domain showed poor antiviral effectiveness and durability. Costimulatory domains like CD28, 4-1BB, or OX40 are incorporated into later second- and third-generation CARs, which show better growth, cytokine production, and in vivo durability [17].

Preclinical research using humanized mouse models has repeatedly demonstrated that HIV-specific CAR-T cells lower viral levels and postpone viral recovery after stopping antiretroviral therapy [18]. However, inadequate trafficking to anatomical reserves reduced efficacy. In order to combat this, CAR-T cells that have been modified to express the chemokine receptor CXCR5 have been established. This allows the cells to home to B-cell follicles, which are a significant refuge for HIV-infected follicular helper T cells (Table 2).

The M10 CAR-T platform, which combines endogenous bNAb release, CXCR5-mediated follicular trafficking, and a bNAb-derived CAR, is a noteworthy therapeutic development. Two infusions of M10 CAR-T cells in conjunction with chidamide-mediated latency activation produced viral rebound suppression in 74.3% of infusions, with an average viral load fall of 67.1% and a 1.15 log_10_ decrease in cell-associated HIV-1 RNA in a phase I trial including 18 HIV-positive people. Crucially, there were no serious treatment-related side effects, indicating that CAR-T-based shock-and-kill tactics are feasible [64].

Compared to CAR-T cells, CAR natural killer (CAR-NK) cells offer a number of benefits as a supplementary cellular immunotherapy platform. Without depending on antigen presentation, NK cells cause cytotoxicity by perforin–granzyme release, death receptor interaction, and antibody-dependent cellular cytotoxicity. Allogeneic donors can produce CAR-NK cells with little chance of graft-versus-host disease, enabling scalable off-the-shelf manufacturing. Preclinical research focusing on HIV envelope proteins has shown that CAR-NK cells have strong antiviral efficacy [65].

### 4.4. Immune Checkpoint Modulation

In persistent HIV infection, immune checkpoint regulation is being investigated as a potential remedy for defective antiviral immunity. T-cell fatigue, a condition marked by decreased cytokine production, poor cytotoxic activity, and decreased proliferative ability, is brought on by prolonged antigen exposure during HIV infection. On HIV-specific CD8+ and CD4+ T cells, this depleted phenotype is linked to persistent elevation of inhibitory receptors, particularly programmed death-1 (PD-1), cytotoxic T-lymphocyte-associated protein 4 (CTLA-4), T cell immunoglobulin and mucin domain-containing protein 3 (TIM-3), lymphocyte activation gene 3 (LAG-3), and TIGIT. Although these checkpoints typically prevent immune-driven tissue damage, their ongoing stimulation in HIV leads to poor viral control and the survival of infected cells [66].

The most thoroughly studied of these mechanisms in HIV is PD-1. Decreased effector function, increased plasma viral loads, and disease progression are all correlated with increased PD-1 expression on HIV-specific CD8 T cells. T-cell receptor signaling is suppressed when PD-1 binds to its ligands, PD-L1 or PD-L2. Preclinical research showed that T-cell proliferation, interferon-γ production, and cytotoxic activity can be partially restored by blocking the PD-1 pathway. PD-1 blockade increased virus-specific T-cell responses and decreased viral load in SIV-infected macaques, but these impacts were short-lived, and viral rebound happened after therapy was stopped, suggesting that checkpoint suppression by itself is not enough for long-term viral control [67,68].

There has not been much clinical research on immune checkpoint inhibitors in HIV-positive individuals, and most of it has focused on patients receiving these drugs for cancer. In the context of suppressive antiretroviral therapy, anti-PD-1 antibodies like pembrolizumab and nivolumab, as well as the anti-CTLA-4 antibody ipilimumab, have typically been delivered safely. In a study, pembrolizumab was used to treat melanoma in an HIV-positive patient on suppressive antiretroviral treatment, temporarily improving HIV-specific CD8+ T-cell activation and activity. There was a slight, transient decrease in total HIV-1 DNA, but other reservoir markers remained unchanged. Overall, the impact of PD-1 blockage alone on the viral reservoir was modest and short-term, indicating the necessity for other latency-reversing techniques [69].

The anti-PD-1 antibody budigalimab achieved sustained PD-1 receptor saturation on CD8+ T cells in a phase 1 trial and was safe and well tolerated in individuals with HIV on antiretroviral therapy. Some subjects experienced temporary off-ART control or a slower viral relapse during the analytical treatment halt, although the effects were inconsistent and exploratory [70].

Checkpoint inhibitors may affect HIV infection in two ways. Checkpoint blockade-induced immunological activation may not only reverse T-cell depletion but also stimulate latent provirus transcription. During checkpoint inhibitor medication, a number of clinical findings have documented brief elevations in plasma HIV RNA, sometimes known as viral blips. These incidents demonstrate the intricacy of checkpoint regulation in the context of viral latency and reservoir dynamics, even if they have not typically led to a loss of viral containment on ART [71].

## 5. Clinical Translation, Ethics, and Regulatory Challenges

Clinical translation of molecular and immunological HIV cure strategies remains a major challenge, constrained by evolving regulatory frameworks, ethical complexity, and persistent scientific uncertainty. Although numerous approaches have demonstrated promising results in preclinical studies, their successful application in humans has been limited by challenges related to model systems, clinical trial design, safety considerations, and scalability. A key obstacle is the limited predictive capacity of current preclinical models. While humanized mouse models provide valuable platforms for mechanistic investigations and proof-of-concept studies, they do not fully replicate human immune responses, tissue architecture, or the spatial distribution and long-term stability of latent HIV reservoirs.

Furthermore, the extreme heterogeneity and widespread distribution of the human HIV reservoir across multiple cellular and anatomical compartments make complete eradication through a single intervention particularly difficult. Viral diversity and persistent immune dysfunction may further reduce the effectiveness of therapeutic strategies in clinical settings. Emerging evidence also indicates that host biological factors contribute to variability in reservoir dynamics. Polymorphisms in genes involved in immune regulation and viral entry, including CCR5 and HLA loci, may influence reservoir establishment, immune recognition of infected cells, and responses to gene-editing or immunotherapeutic interventions. In addition, sex-based hormonal and immunological differences have been associated with variations in immune activation and viral transcriptional activity, potentially affecting reservoir persistence and responsiveness to cure-directed strategies. Although SIV or SHIV-based non-human primate models more closely resemble features of HIV pathogenesis and immune regulation, they are nonetheless expensive, scarce, and biologically different from human infection. Extrapolating safety and effectiveness information to clinical settings is made more difficult by these limitations [72]. The scarce nature and variability of latently infected cells hamper the evaluation of curative options in clinical trials. Large sample sizes and extended follow-up are necessary because sensitive tests with inherent variability are needed to measure reservoir size accurately and identify slight intervention-induced changes. Analytical treatment interruption (ATI) has been used in numerous trials due to the lack of defined surrogate endpoints that accurately predict sustained post-treatment viral control. Although ATI has become the most direct way to measure efficacy, there are hazards associated with it, such as HIV transmission, immunological activation, viral rebound, and possible reservoir enlargement. Therefore, strict participant selection, close monitoring, predetermined ART restart criteria, and risk reduction techniques are necessary for ATI research to be conducted ethically [73].

Cellular treatments and gene editing provide unique safety and regulatory issues. Because inadvertent genetic changes may result in genomic instability or disruption of vital host genes, off-target genome editing continues to be a major problem. Even though specificity has increased due to advancements in editing technology, thorough genomic evaluation and long-term patient monitoring are crucial. Although regulatory bodies have set up procedures for gene and cell therapies, it is still unclear what clinical objectives, follow-up time, and efficacy parameters are suitable in the context of HIV cure. Ethical issues are especially important because patients in cure trials are usually virologically suppressed on antiretroviral therapy and clinically stable. As a result, patients may be at risk and have no immediate clinical benefit. The experimental aspect of therapies, the poor chance of recovery with existing approaches, and the dangers of stopping therapy must all be made abundantly known in informed consent procedures. It is an ongoing issue to make sure that involvement in research does not unfairly burden vulnerable communities or worsen global health disparities [74,75]. Lastly, cost and scalability are major barriers to wider adoption. Autologous cell-based treatments are not feasible in low- and middle-income environments due to their complicated, resource-intensive production procedures. Furthermore, a lot of cutting-edge interventions, such as gene-editing techniques and CAR-T cell therapies, need highly controlled production processes, specialized laboratory infrastructure, and dependable cold-chain distribution, all of which are frequently lacking in low-resource healthcare settings. Large-scale adoption may be hampered by these financial and logistical issues, which emphasizes the significance of creating therapeutic approaches that are both straightforward and scalable. On the other hand, long-acting antiretroviral drugs, therapeutic vaccinations, and broadly neutralizing antibodies might provide more scalable solutions, although infrastructure, pricing, and distribution issues still exist. For HIV cure research to be responsibly and fairly translated into clinical application, these scientific, ethical, and legal obstacles must be addressed [76,77].

## 6. Future Directions

Future developments in our knowledge of reservoir biology, technical advancements, and practical therapeutic goals will determine the course of HIV cure research. Clarifying the mechanisms controlling the latent reservoir’s formation, maintenance, and heterogeneity is a top objective. There are still unanswered questions about why some cellular subsets and anatomical locations allow for long-term viral persistence while others allow for degradation. In order to find reservoir cell morphologies, proviral traits, and the tissue microenvironments that encourage latency, single-cell multi-omics techniques, spatial transcriptomics, and high-dimensional imaging are being used more frequently. These techniques provide information to facilitate more focused interventions [78].

Gene-based therapy technological advancements are another crucial field for further growth. Next-generation gene editing tools, including base and prime editors, offer better selectivity and lower risk of genotoxicity compared with prior double-strand break-inducing techniques. The discovery of RNA-targeting CRISPR systems and compact Cas variations broadens the pool of possible therapeutic targets. However, advancements in delivery technologies—such as modified viral vectors, lipid nanoparticles, virus-like particles, and extracellular vesicles—that can provide potent and tissue-dependent targeting of reservoir cells will determine how effective these tools are [79].

The advancement of cure research still depends on improving the latent reservoir’s measurement and characterization capabilities. The sensitivity and scalability of current assays are restricted, and they are unable to accurately predict viral management after treatment. The development of standardized tests that precisely measure replication-competent virus, differentiate intact from defective proviruses, and evaluate reservoir distribution across tissues will be the main focus of future work. Personalized treatment tactics and more effective clinical trial designs may be made possible by the discovery and confirmation of immunological and virological indicators linked to post-treatment control. It is anticipated that computational methods will become more important in directing future investigations. Mathematical modeling of viral dynamics and immune reactions can guide the design and sequencing of combined treatments, while artificial intelligence and machine learning techniques can facilitate examination of intricate datasets, determination of therapy targets, and optimization of immunotherapeutic constructs. Lastly, worldwide viability must be taken into consideration in future treatment plans. Many sophisticated therapies are nevertheless expensive and complicated, which restricts their use in environments with low resources. The development of scalable, cheap techniques that can be deployed across varied healthcare systems will be vital for having a real worldwide effect [80,81,82]. Furthermore, current debates on HIV cure target product profiles emphasize how crucial it is to take patient preferences, acceptable risk–benefit profiles, and practical delivery methods into account when developing future cure efforts. By taking these factors into account in the early stages of research and clinical development, it may be possible to guarantee that new interventions continue to work with a variety of patient populations and healthcare environments.

## 7. Conclusions

HIV cure research has progressed via small but significant strides in molecular and immunological approaches aimed at tackling viral latency and reservoir persistence. Recent research shows that gene editing tools, especially CRISPR-based techniques, can disrupt proviral DNA or host entry factors in preclinical and early clinical contexts; nevertheless, clinical application is still restricted by delivery and safety issues. Although latency-directed therapies have improved our understanding of HIV transcriptional regulation, they have not consistently reduced the size of the reservoir in human trials. Whilst immunological methods, such as CAR-T cell treatments and broadly neutralizing antibodies, have demonstrated quantifiable impacts on viral suppression and delayed rebound after analytical treatment interruption, they are not sufficient to establish long-term control on their own. All of these results point to significant biological obstacles to HIV elimination. Continued rigorous assessment of these approaches, bolstered by improved biomarkers and meticulously designed clinical trials, will be key to moving HIV cure investigations beyond suppressive antiretroviral therapy.

## Figures and Tables

**Table 1:** Molecular innovations for HIV treatment.

Technique	Target(s)	Mechanism of Action	Model/System	Key Findings	Reference
Combinatorial CRISPR-Cas9 editing of both host and viral genomes	CCR5 co-receptor; integrated HIV-1 provirus	Destroy CCR5 entrance receptor and remove proviral DNA after ART	ART-suppressed humanized mice	In about 58% of mice, dual editing resulted in the long-term eradication of replication-competent HIV in various tissue compartments; no resurgence in virus was observed; and CD4+ restoration was enhanced.	[34]
CRISPR-Cas9 ribonucleoprotein (RNP)-mediated dual gene disruption	CCR5 and CXCR4 entry co-receptors	Ex vivo CRISPR-Cas9 RNP administration decreases CCR5 & CXCR4 expression, inhibiting R5, X4, and dual-tropic HIV penetration	Human T cells & humanized mice	Dual disruption substantially decreased surface CCR5/CXCR4 and developed resistance to CCR5-tropic, CXCR4-tropic, and dual-tropic HIV-1 strains; modified cells demonstrated longevity benefit in vivo.	[35]
CRISPR-Cas9 genome editing with gRNAs targeting LTR regions	HIV-1 integranted proviral DNA (5′ and 3′ LTR-flanking sequences; internal regions)	CRISPR-Cas9 fragmentation at proviral LTR targets eliminates or destroys integrated HIV DNA and lowers expression	Latently infected human CD4+ T-cells (cell lines)	Targeted LTR breakage resulted in elimination or disruption of proviral DNA between LTRs and substantially decreased HIV expression; engineered cells resisted reactivation and new infection.	[36]
CRISPR/dCas9-KRAB mediated repression of proviral LTR	HIV-1 LTR promoter regions (NF-κB binding sites)	Chromatin repressors are recruited at LTRs by dCas9 coupled to KRAB repressor to prevent transcription.	Latently infected T (J-Lat 10.6) and U1 myeloid cell models	Specific sgRNAs focusing on LTR sequences with dCas9-KRAB strongly inhibited HIV-1 reactivation caused by PMA, SAHA, and other stimuli; additionally, up to ~100-fold decrease in viral RNA expression was observed	[37]
siRNA and shRNA-mediated RNA interference targeting viral RNA	HIV-1 Gag mRNA (highly conserved site)	Viral mRNA is broken down by sequence-specific RNA interference, which lowers the synthesis of viral proteins and virions.	HEK293T cells cotransfected with HIV-1 molecular clone	Dicer substrate siRNAs (27–29 nt) and improved shRNA designs substantially blocked HIV-1 production more efficiently than canonical formats.	[38]
mRNA-Lipid Nanoparticle (LNP-X) delivery of HIV Tat mRNA	HIV transcriptional activator Tat protein (encoded by delivered mRNA)	Tat mRNA is delivered by LNP-X to CD4+ T cells that are at rest. Translated Tat increases HIV transcription and reverses latency.	Ex vivo resting CD4+ T cells	Effective mRNA administration allows for the control of both viral and host gene expression and causes HIV transcriptional activation (latent reservoir reactivation) without toxicity or widespread T-cell activation.	[39]
Long-acting small-molecule (Lenacapavir) HIV capsid inhibitor added to optimized background regimen	HIV-1 capsid protein (CA)	Clings to the HIV-1 capsid, strengthening the capsid lattice to prevent nuclear import of viral DNA, interrupting uncoating, and meddling with the last phases of virion maturation.	Adults with multidrug-resistant HIV-1 in a randomized phase 2/3 clinical trial	At week 52, 83% of subjects attained HIV-1 RNA < 50 copies/mL, suggesting significant antiviral effectiveness of lenacapavir in heavily treatment-experienced persons, with typically acceptable tolerability despite some apparent resistance.	[40]
Small-molecule HIV-1 capsid inhibitor (GS-CA1)	HIV-1 capsid protein (CA)	Attaches with HIV-1 capsid with ultrapotent capacity, upsetting orderly capsid assembly and particle generation in addition to inhibiting nuclear import, leading to robust suppression of replication	Humanized mouse model	Capsid inhibitor can effectively inhibit HIV-2 and all major HIV-1 varieties, such as viral variants resistant to ARVs presently in clinical usage. GS-CA1 demonstrated greater antiviral efficiency as a long-acting injectable monotherapy in a humanized mouse model of HIV-1 infection, surpassing long-acting rilpivirine.	[41]

**Table 2:** Immunological Innovations for HIV Control.

Technique	Target(s)	Mechanism of Action	Model/System	Key Findings	Reference
Triple bNAb cocktail (PGT121, PGDM1400, VRC07-523LS) infusions	HIV-1 Env conserved epitopes (V2 apex, V3 glycan, CD4bs)	Neutralizes a variety of viral strains & lowers viremia by inhibiting entry/evasion	Adults with HIV during ART interruption	83% of patients retained virologic suppression for at least 28 weeks, and 42% did so for 38–44 weeks despite decreasing bNAb amounts, with correlates of decreased immune stimulation and depletion	[59]
bNAb-derived CAR T cell adoptive therapy	CAR targeting HIV-1 Env on infected cells	Cytotoxic eradication of HIV-expressing cells	Phase I open-label human trial	HIV-1 patients responded favorably to bNAb-derived CAR T cell treatment, which also markedly decreased intact proviral reservoirs and cell-associated viral RNA. However, preexisting or newly emergent escape mutations under CAR T cell-mediated immunological pressure caused a viral resurgence following ART interruption.	[60]
mRNA-encoded, membrane-anchored HIV Env trimer vaccine	HIV-1 Env trimer antigens (gp140/151)	Evokes Env-specific B-cell and neutralizes antibody reactions	Phase 1 clinical trial	In 80% of patients, membrane-anchored mRNA Env trimers produced tier 2 autologous neutralizing antibodies, demonstrating high immunogenicity and tolerance.	[61]
Anti-HIV CAR-T cells combined with low-dose rapamycin	HIV-specific CAR-T cells (derived from HSCs) and pathways regulating exhaustion (e.g., PD-1, Tim-3 inhibitory circuits)	Rapamycin decreases long-term inflammation, avoids depletion of CAR-T cells, and enhances CAR-T control of viral replication	HIV-infected humanized mice	Compared to CAR-T alone, rapamycin + CAR-T enhanced immunological control, decreased the HIV reservoir (reduced HIV DNA/RNA levels), and prolonged viral relapse following ART cessation.	[62]
CTLA-4 blockade during HIV virus-like particle (VLP) immunization	CTLA-4 on regulatory T cells; Env-specific Tfh and B-cell subsets	CTLA-4 inhibition improves antigen-specific Tfh help, class switching, and high-affinity IgG.	HIV VLP immunization in mice	Env- and Gag-specific IgG levels were markedly raised by CTLA-4 blockade + VLPs, which also expedited class switching, raised APRIL and IL-21 signals, and improved ADCC.	[63]

## Data Availability

No new data were created or analyzed in this study.
